# Association of Serum 25-Hydroxyvitamin D with Life Style and Dietary Factors in Egyptian Prepubescent Children

**DOI:** 10.3889/oamjms.2015.006

**Published:** 2015-01-01

**Authors:** Mones M. Abu Shady, Mai M. Youssef, Manal A. Shehata, Ebtissam M. Salah El-Din, Heba A. ElMalt

**Affiliations:** 1 *Child Health Department, National Research Centre, Cairo, Egypt*; 2 *Medical Biochemistry Department, Medical Division, National Research Centre, Cairo, Egypt*

**Keywords:** 25-hydroxyvitamin D, lifestyle, physical activity, BMI, children

## Abstract

**BACKGROUND:**

There had been a growing evidence of high prevalence of vitamin D deficiency especially among children which may increase the risk of many chronic diseases in adulthood.

**AIM:**

Assessment of different lifestyles and dietary behaviour influencing the level of serum 25-OHD in a group of Egyptian prepubescent children.

**SUBJECTS AND METHODS:**

Two hundred boys and girls aged from 9 to 11 years were recruited from two primary public schools situated in Giza governorate in Egypt. A questionnaire was developed to obtain relevant information related to age, dietary habits, and physical activity. Thorough clinical examination and measurement of weight and height were performed. Body mass index was calculated. Serum samples were assayed for 25-hydroxy vitamin D (25-OHD).

**RESULTS:**

Low serum 25-OHD (< 20 ng/ml) was found in 11.5% of the whole sample. Mean serum 25-OHD was significantly lower in obese subjects and in those with low physical activity (p < 0.05). Multiple stepwise linear regression analysis showed that BMI and physical activity were the main predictors of serum 25-OHD (P < 0.05).

**CONCLUSIONS:**

Lifestyle factors in terms of physical activity and BMI may contribute significantly to the optimal vitamin D status of apparently healthy children.

## Introduction

The prevalence of vitamin D deficiency has increased rapidly worldwide in both children and adults during the past decade [1]. Vitamin D insufficiency affects almost 50% of the population worldwide. An estimated 1 billion people worldwide, across all ethnicities and age groups, have a vitamin D deficiency (VDD). It is common in Australia, the Middle East, India, Africa, and South America [2].

Vitamin D is produced in the skin upon ultraviolet B exposure, acting on 7-dehydrocholesterol and undergoing hydroxylation in the liver to 25-hydroxyvitamin D (25-OHD) and kidneys to 1,25- dihydroxyvitamin D [3]. The plasma concentration of 25-OHD is the most commonly used biomarker of vitamin D status because it has a long half-life and because it is not under tight homeostatic regulation and therefore reflects vitamin D supply and usage over a period of time [4].

Vitamin D deficiency is attributed to a variety of causes including restriction of sunlight exposure (low sun exposure - clothing due to religious practices), deficient consumption of vitamin D-rich products, atmospheric pollution, geographic latitude and altitude and malabsorption of vitamin D [5].

Low serum 25-OHD level has been associated with several diseases including rickets, osteomalacia, cancer (colon, breast, prostate), type 2 diabetes, osteoporosis, autoimmune diseases (type 1 diabetes, inflammatory bowel disease, multiple sclerosis), cardiovascular diseases and metabolic syndrome [6].

Regular physical activity plays a significant role in enhancing and maintaining human health. However, the contribution of school-based physical education remains unacceptably low, and the time spent in free play is becoming increasingly scarce [7]. For the prevention of vitamin D deficiency, healthy life style with outdoor activities and associated sun exposure and intake of fortified nutrition should be advised [8]. The new Dietary Reference Intakes (DRI) increased greatly the recommended dietary allowances (RDA) of vitamin D from 200 to 600 IU/d (15 μg/d) for individuals from 1 to 70 years of age [9].

The purpose of this study was to investigate the role of different lifestyles and dietary behaviours in influencing the level of serum 25-OHD in a group of Egyptian prepubescent children.

## Subjects and Methods

A cross-sectional study comprised 200 children (98 boys and 102 girls). They were Prepubescent (Tanner stage 1) aged from 9 to 11 years. The pupils were recruited from two primary public schools situated in Giza governorate in Egypt. Exclusion criteria were: age of children was below 9 or over 11years, if children had endocrinal or genetic obesity, or had chronic debilitating diseases: e.g. (hepatic or renal disease, diabetes mellitus, rheumatic and congenital heart diseases, hypertension and chronic lung diseases), or with metabolic diseases (metabolic rickets, calcium metabolism disorders), mal-absorptive disorders (Crohn’s disease, cystic fibrosis, and celiac disease) and cancer and if they have taken medications as anticonvulsants or systemic glucocorticoids or those taking calcium, vitamin D or multivitamin supplements.

The children were asked about frequency of milk intake/week and frequency of eating fast food. The questionnaire also sought information about sun exposure (frequency of exposure, duration of exposure) and physical activity, which is self-reported (frequency and type of activities performed along with duration – number of minutes per week) and hours of watching TV.

Written informed consents were obtained from parents after explanation the aim of the study. The study was approved by the medical ethics committee of the National Research Centre, Cairo, Egypt.

### Physical Examination

Children were subjected to thorough clinical examination that included chest, heart, abdominal, and central nervous system examination. Assessment of pubertal development was according to Tanner’s scoring [10].

The Anthropometric measurements, that include: weight, height, were performed. The height was measured to the nearest 0.1 cm using a Holtain portable anthropometer, and the weight was determined to the nearest 0.01 kg using a Seca scale Balance with the subject dressed in minimal clothes and without shoes. The BMI was calculated as weight (in kilograms) divided by height (in meters) squared. BMI- Z-score was calculated based on the WHO growth standards [11] with the help of Anthro-plus Program for personal computers. Children with BMI- Z-score >2 were considered obese [11].

### Determination of 25-OHD

A venous blood sample was withdrawn from each child after an overnight fast of 12 hr. Serum was separated and stored at –20°C until assayed. Serum samples were assayed for 25-OHD using enzyme linked immunosorbent Assay (ELISA) kit, provided by Immunodiagnostic Systems GmbH, Germany [12]. Serum 25-OHD is the major circulating form of vitamin D and a standard indicator of vitamin D status [13]. A concentration of less than 20 ng/mL, is considered an indication of vitamin D deficiency [1].

### Statistical analysis

Statistical analysis was carried out using the statistical package for social sciences, version 16 for windows (SPSS Inc., USA). Continuous data were expressed as mean ± SD and were compared using Student’s t-test. Categorical data were expressed as frequencies and percentages. Pearson’s correlation analysis was carried out to evaluate the association between continuous exposure and continuous covariates. Simple linear regression and multiple stepwise regression analyses were used to identify the significant predictive covariates of serum 25-OHD concentrations. P value less than 0.05 was considered as statistically significant.

## Results

The mean age of the whole subjects studied was 10.39 ± 0.58 years (age ranged from 9-11 years). Of them, 98 were males (49%) and 102 were females (51%). Obese subjects (BMI z-score > 2) were 88 (44%) and non-obese subjects were 112 (56%). Low serum 25-OHD (< 20 ng/ml) was found in 23 subjects (11.5%) while the remaining 177 subjects (89.3%) had adequate serum 25-OHD.

Table 1 shows the mean serum level of 25-OHD according to gender, BMI, physical activity and nutritional habits. There was no significant differences detected in mean serum 25-OHD regarding gender and fast food intake (p > 0.05). While it was significantly lower in obese subjects than non-obese and in those with low physical activity than those with high physical activity (p < 0.05). According to nutritional habits, mean serum 25-OHD was significantly lower in those who drink milk less than once/day than in those who drink it once or more/day (p < 0.05).

**Table 1:** Mean 25-OHD concentrations by sex, body mass index, physical activity and dietary habits.

	Number	25-OHD (ng/ml)	t-test	p

		Mean ± SD		
Sex				
Male	98	39.46 ± 12.39	-1.72	0.087
Female	102	43.57 ± 14.19		

BMI				
Obese	88	32.41 ± 12.75	-10.56	0.000*
Non-obese	112	48.74 ± 9.11		

Physical activity				
Low (<4 hours/week)	131	38.61 ± 13.29	-4.425	0.000*
High (≥4 hours/week)	69	47.14 ± 12.28		

Milk intake				
Less than once/day	72	36.11 ± 14.69	-4.47	0.000*
Once or more/day	128	44.62 ± 11.85		

Fast food intake				
>Once/week	134	43.56 ± 14.26	1.52	0.132
Once or less /week	66	40.06 ± 12.63		

* P < 0.05 is significant; BMI = Body mass index.

Table 2 shows correlation between serum 25-OHD and different life style and nutritional parameters. Serum 25-OHD was significantly correlated with BMI, physical activity and milk intake/week (p < 0.05). Hours of watching TV and sun exposure and frequency of eating fast food were not significantly correlated with serum 25-OHD (p > 0.05).

**Table 2:** Correlation between serum 25-OHD and different life style and nutritional parameters.

	25-OHD	
	r	p
BMI	-0.736	0.000*
Hours of physical activity/week	0.306	0.000*
Hours watching TV/day	0.002	0.980
Hours of sun exposure/day	-0.132	0.111
Frequency of eating fast food/week	0.124	0.132
Frequency of milk intake/week	0.200	0.039*

* P < 0.05 is significant; BMI = Body mass index

Simple linear regression analysis was done to confirm the predictors of serum 25-OHD (Table 3). It showed that BMI, physical activity and milk consumptions were the main predictors of serum 25-OHD. Further analysis through multiple stepwise linear regression showed that BMI and physical activity alone were the main predictors of serum 25-OHD (P < 0.05).

**Table 3:** Predictors of 25-OHD.

	Simple linear regression			Multiple linear regression		
Variable	Βcoefficient	R^2^	p	Βcoefficient	R^2^	p

BMI	-0.736	0.541	0.000*	-1.980	0.734	0.000*

Physical activity (hours/week)	0.306	0.093	0.000*	0.336	0.806	0.000*

Frequency of milk intake/week	0.200	0.040	0.039*			

Frequency of eating fast food/week	0.124	0.015	0.132			

* P < 0.05 is significant; BMI = Body mass index.

## Discussion

In the present study the main predictors of serum 25-OHD were BMI and physical activity. Mean serum 25-OHD was significantly higher in non-obese subjects compared with the obese. Moreover, subjects with high physical activity had significant higher serum 25-OHD than those with low physical activity. Although subjects who had milk intake once or more/day, had significant higher serum 25-OHD but multiple linear regression analysis did not confirm this significance.

In the current study we found an inverse association between 25OHD level and BMI. Association of low 25-OHD level and obesity has been seen in several studies [14-17]. It is not known whether low 25-OHD level is due to for example changes in vitamin D metabolism in obese individuals or life style factors associated with obesity, for example low sun light exposure. It is known that 25-OHD is stored in adipose tissue and muscle. The sequestration of vitamin D into adipose tissue has been proposed to explain this association. It is possible that bioavailability of 25-OHD or its precursors stored in adipose tissue is poor [18]. Same results were detected in a study done in Al Ain, Abu Dhabi Emirate, which found an inverse correlation between serum 25-OHD concentrations with BMI and positive correlation with physical activity after adjustment for age [19]. In contrast to the present study, they found inverse correlation between serum 25-OHD with female gender and consumption of fast food per week. That difference may be due to the different age group (12-18 years) selected in their study. In studies by dong et al., Sioen et al., and Valtuena et al., [20-22], independent influence of BMI, physical fitness, seasonal variation on serum 25-OHD concentrations was detected.

Consistent with our findings, Brock et al., [23], reported that the major modifiable predictors of low vitamin D status were body mass index (BMI) >30 kg/m^2^, physical inactivity in addition to calcium supplement intake. Same results were detected in sunny areas like Saudi Arabia, where vitamin D deficiency is common among Saudi children and adolescents, and is influenced by both sun exposure and physical activity. Promotion of an active outdoor lifestyle among Saudi children in both homes and schools may counteract the vitamin D deficiency epidemic in this vulnerable population [24]. A study in Republic of Korea postulated that an increase in vigorous PA and vitamin D intake should be two major targets of public health inventions against the clustering of metabolic risk factors in the Korean pediatric population [25].

It is well established that physical activity increases local bone mass, reduces calcium excretion and raises absorption efficiency, thus increasing serum calcium which results in sparing serum vitamin D. In addition, physical activity which is known to reduce body weight by increasing the rate of lipolysis may enhance mobilization of vitamin D from adipose tissue, thus increasing its serum level [26, 27]. Recently it has been suggested that muscle provides an extravascular pool through which 25OHD circulates. This could explain the positive association between physical activity and 25-OHD level [28, 29].

However, frequency of fast food intake was not significantly linked with vitamin D levels. Fortified milk could be the most important source of vitamin D. Previous studies have associated milk consumption with higher 25-OHD levels [30, 31]. Similarly the present study found positive significant correlation between the frequency of milk intake and 25-OHD levels. Jang et al., [32] in their study, found that healthy dietary habits, such as consuming more milk and fewer soft drinks, could be helpful to achieve an adequate vitamin D level.

Other possible determinants of low vitamin D status could be low sunshine exposure which can be explained by lower body surface area (BSA) exposed, less time spent on outdoor physical activity and greater indulgence in indoor activities like watching television, computer gaming, indoor tuition and other recreational activities among children [33-35]. Vitamin D status was investigated in adolescent females (14-17 years) in Egypt and it was found that, both sun exposure index and daily sun exposure time were significantly higher in girls with adequate vitamin D levels compared to those with insufficient and deficient vitamin D. Exposure of at least 18% of BSA for at least 37 minutes/day is enough to achieve adequate vitamin D levels in a sunny climate as Egypt [36]. In the present study, sun exposure was not a predictive variable of serum 25-OHD concentration and that difference from the previous study may be attributed to the different age group studied and the different seasons during which the studies were carried out.

Our study acknowledges a few limitations. The study was cross-sectional, and therefore, causality cannot be inferred. Information on sun exposure, dietary and physical activity information were based on administered questionnaires which are subject to recall bias. We did not evaluate the effect of seasonality as the study was conducted during winter months. Given the availability of sun shine almost throughout the year the seasonality may not be an important factor. Our results may not be generalizable as the sample size was small.

In conclusion, vitamin D deficiency was prevalent in 11.5% of the studied sample. Lifestyle factors in terms of physical activity and BMI may contribute significantly to the optimal vitamin D status of apparently healthy children. Given the potential for serious morbidity, there is a need for urgent monitoring of vitamin D deficiency and timely correction of vitamin D status in the Egyptian population.
